# *catena*-Poly[[[bis­(*O*,*O*′-diisobutyl dithio­phosphato-κ^2^ *S*,*S*′)nickel(II)]-μ-1,2-bis­(4-pyridylmethyl­ene)hydrazine-κ^2^ *N*:*N*′] toluene disolvate]

**DOI:** 10.1107/S1600536808017121

**Published:** 2008-06-13

**Authors:** Erick Berdugo, Edward R. T. Tiekink

**Affiliations:** a Department of Chemistry, The University of Texas at San Antonio, One UTSA Circle, San Antonio, Texas 78249-0698, USA

## Abstract

The polymeric title compound, {[Ni(C_8_H_18_O_2_PS_2_)_2_(C_12_H_10_N_4_)]·2C_7_H_7_}_*n*_, has a linear topology and features octa­hedrally coordinated Ni atoms with a *trans*-N_2_S_4_ donor set. The toluene solvent mol­ecules occupy channels defined by the three-dimensional stacking of the polymeric chains. The Ni atom is located at a centre of inversion and the bridging 1,2-bis­(4-pyridylmethyl­ene)hydrazine4-pyridine mol­ecule is also disposed about a centre of inversion. One isobutoxy group is disordered equally over two positions.

## Related literature

For a related structure, see: Berdugo *et al.* (2007 ▶). For related literature, see: Lai *et al.* (2004 ▶); Chen *et al.* (2006 ▶); Tiekink (2006 ▶); Benson *et al.* (2007 ▶).
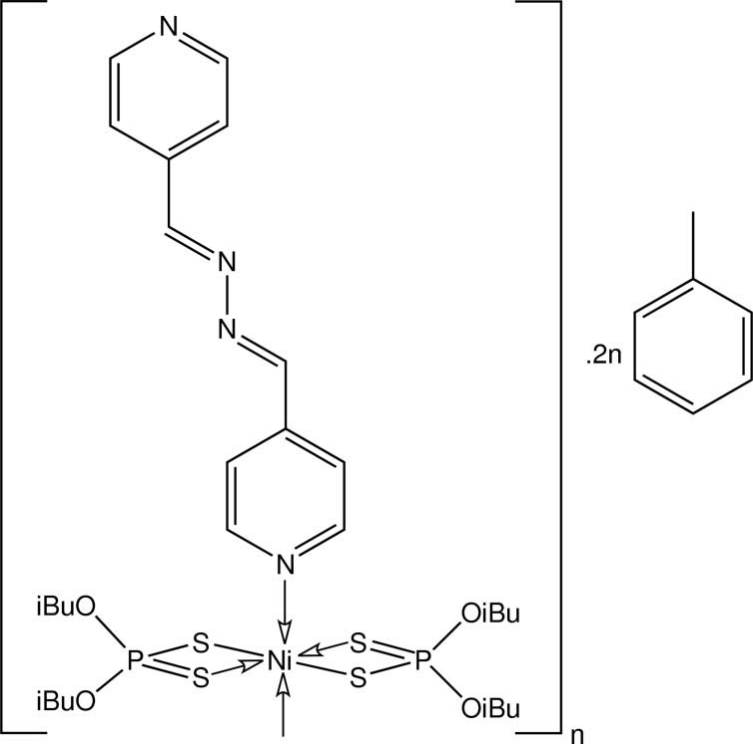


## Experimental

### 

#### Crystal data

[Ni(C_8_H_18_O_2_PS_2_)_2_(C_12_H_10_N_4_)]·2C_7_H_7_
*M* *_r_* = 935.85
Triclinic, 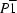

*a* = 8.7132 (15) Å
*b* = 12.089 (2) Å
*c* = 12.293 (2) Å
α = 82.662 (10)°
β = 86.528 (10)°
γ = 69.321 (6)°
*V* = 1201.4 (4) Å^3^
*Z* = 1
Mo *K*α radiation
μ = 0.69 mm^−1^
*T* = 98 (2) K
0.30 × 0.20 × 0.10 mm

#### Data collection

Rigaku AFC12K/SATURN724 diffractometer
Absorption correction: multi-scan (*ABSCOR*; Higashi, 1995 ▶) *T*_min_ = 0.795, *T*_max_ = 1 (expected range = 0.742–0.934)
8239 measured reflections
5457 independent reflections
4980 reflections with *I* > 2σ(*I*)
*R*_int_ = 0.033

#### Refinement

*R*[*F* ^2^ > 2σ(*F* ^2^)] = 0.054
*wR*(*F* ^2^) = 0.142
*S* = 1.08
5457 reflections
279 parameters
H-atom parameters constrained
Δρ_max_ = 0.64 e Å^−3^
Δρ_min_ = −0.78 e Å^−3^

### 

Data collection: *CrystalClear* (Rigaku, 2005 ▶); cell refinement: *CrystalClear*; data reduction: *CrystalClear*; program(s) used to solve structure: *SHELXS97* (Sheldrick, 2008 ▶); program(s) used to refine structure: *SHELXL97* (Sheldrick, 2008 ▶); molecular graphics: *ORTEPII* (Johnson, 1976 ▶) and *DIAMOND* (Brandenburg, 2006 ▶); software used to prepare material for publication: *SHELXL97*.

## Supplementary Material

Crystal structure: contains datablocks global, I. DOI: 10.1107/S1600536808017121/ng2459sup1.cif

Structure factors: contains datablocks I. DOI: 10.1107/S1600536808017121/ng2459Isup2.hkl

Additional supplementary materials:  crystallographic information; 3D view; checkCIF report

## Figures and Tables

**Table 1:** Hydrogen-bond geometry (Å, °)

*D*—H⋯*A*	*D*—H	H⋯*A*	*D*⋯*A*	*D*—H⋯*A*
C12—H12⋯S1^i^	0.95	2.76	3.694 (3)	169

Symmetry code: (i) 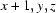.

**Fig. 1.**
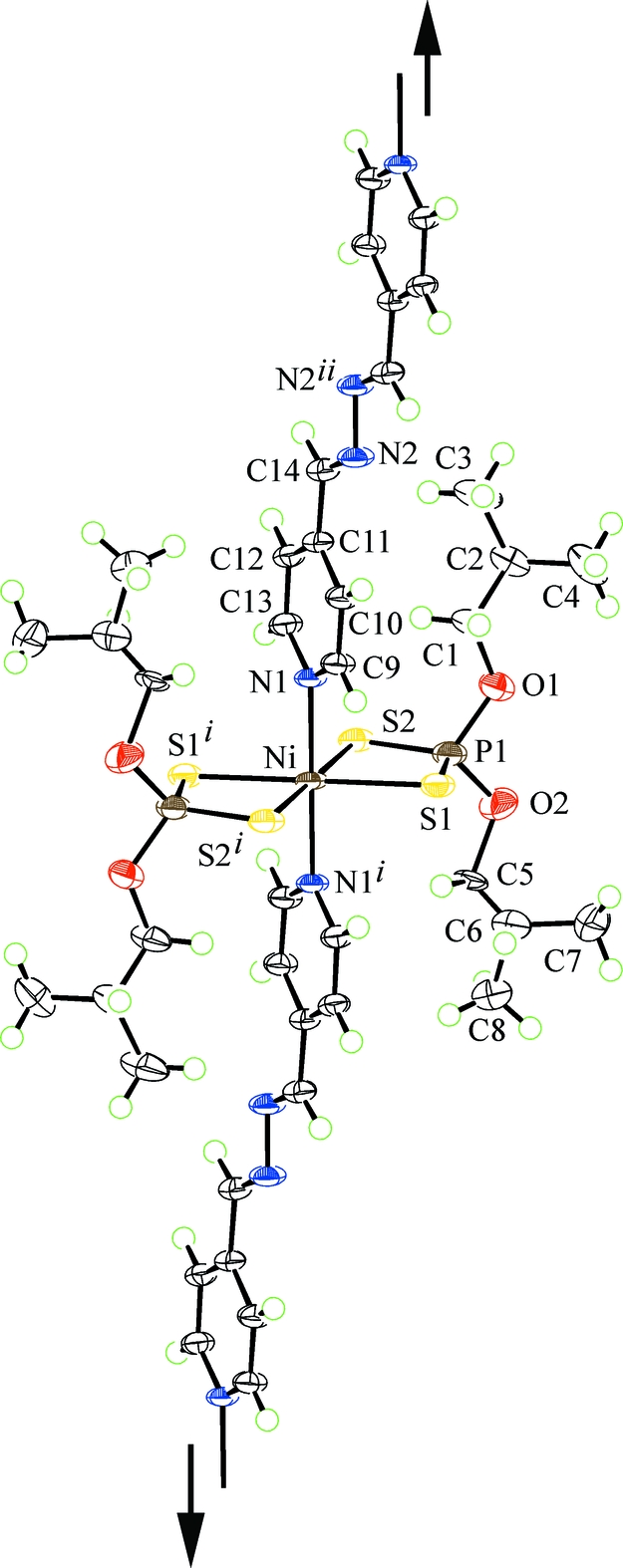
Coordination geometry of the Ni atom in (I) showing the crystallographic numbering scheme. Displacement ellipsoids are shown at the 70% probability level. Symmetry operation i: 1 - x, 1 - y, 1 - z & ii: 2 - x, 1 - y, 2 - z. Only one orientation of the disordered O2—C5—C8 butyl group is shown for clarity.

**Fig. 2.**
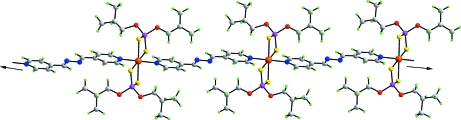
Polymer topology in (I). Colour code: Ni (orange), S (yellow), P (pink), O (red), N (blue), C (grey) & H (green).

**Fig. 3.**
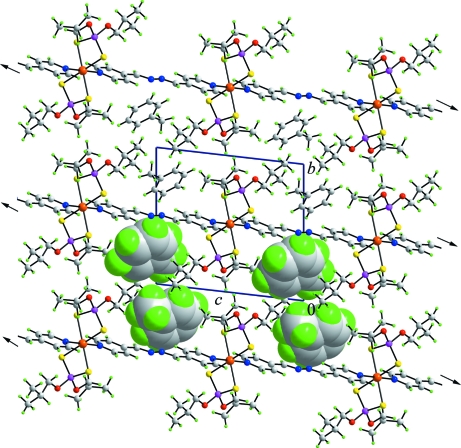
A view of the crystal packing in (I) highlighting the channels occupied by the solvent toluene molecules (four shown in space filling mode). Colour code as for Fig. 2.

## supplementary crystallographic information

### Comment

Interest in examining structures related to the title compound (I), Fig. 1, relates in the main to attempts to control polymer formation and when formed topology (Lai *et al.*, 2004; Chen *et al.* 2006; Tiekink, 2006; Benson *et al.*, 2007 & Berdugo *et al.*, 2007). A linear polymer is found in (I), Fig. 2, in which the Ni atom is located on a centre of inversion and the bridging 4-pyridinealdazine ligand is disposed about another centre of inversion. The Ni atom exists in a *trans*-N_2_S_4_ octahedral coordination geommetry. The polymers are aligned along the c-direction and form layers in the *ac*-plane that are stabilized by C—H···S contacts, Table 1. Layers stack along the *b* axis and define approximate squares with Ni···Ni edges 12.1 and 12.3 Å. Despite the fact that the isobutyl residues protrude into the resulting channels, the toluene molecules are accommodated in these as seen in Fig. 3.

### Experimental

The title compound was prepared by refluxing equimolar amounts of the parent nickel dithiophosphate with 4-pyridinealdazine (Sigma-Aldrich) in toluene (30 ml) for 30 min following a literature procedure (Berdugo *et al.*, 2007). Brown crystals of (I) were isolated by the slow evaporation (3 days) of this toluene solution. The crystals lost crystallinity with standing in air after a few minutes. IR (cm^-1^): v(C—O) 1126, v(P—O) 951, v(P—S)_asymm_ 672, v(P—S)*_sym_* 593.

### Refinement

The methyene-C5 and methine-C6 atoms of the O2—C5—C8 butyl group were disordered over two sites with s.o.f. = 0.5 (from anisotropic refinement); the O2, C7 and C8 atoms were localized in one site only. The H atoms were geometrically placed (C—H = 0.95–1.00 Å) and refined as riding with *U*_iso_(H) = 1.2*U*_eq_(C) or 1.5*U*_eq_(methyl C).

### Crystal data

**Table:**

[Ni(C_8_H_18_O_2_PS_2_)_2_(C_12_H_10_N_4_)]·2C_7_H_7_	*Z* = 1
*M**_r_* = 935.85	*F*(000) = 496
Triclinic, *P*1	*D*_x_ = 1.294 Mg m^−^^3^
Hall symbol: -P 1	Mo *K*α radiation, λ = 0.71070 Å
*a* = 8.7132 (15) Å	Cell parameters from 4131 reflections
*b* = 12.089 (2) Å	θ = 2.3–40.7°
*c* = 12.293 (2) Å	µ = 0.69 mm^−^^1^
α = 82.662 (10)°	*T* = 98 K
β = 86.528 (10)°	Prism, brown-orange
γ = 69.321 (6)°	0.30 × 0.20 × 0.10 mm
*V* = 1201.4 (4) Å^3^	

### Data collection

**Table:**

Rigaku AFC12K/SATURN724 diffractometer	5457 independent reflections
Radiation source: fine-focus sealed tube	4980 reflections with *I* > 2σ(*I*)
graphite	*R*_int_ = 0.033
ω scans	θ_max_ = 27.5°, θ_min_ = 2.3°
Absorption correction: multi-scan (*ABSCOR*; Higashi, 1995)	*h* = −11→11
*T*_min_ = 0.795, *T*_max_ = 1	*k* = −13→15
8239 measured reflections	*l* = −15→15

### Refinement

**Table:**

Refinement on *F*^2^	Primary atom site location: structure-invariant direct methods
Least-squares matrix: full	Secondary atom site location: difference Fourier map
*R*[*F*^2^ > 2σ(*F*^2^)] = 0.054	Hydrogen site location: inferred from neighbouring sites
*wR*(*F*^2^) = 0.142	H-atom parameters constrained
*S* = 1.08	*w* = 1/[σ^2^(*F*_o_^2^) + (0.064*P*)^2^ + 1.4047*P*] where *P* = (*F*_o_^2^ + 2*F*_c_^2^)/3
5457 reflections	(Δ/σ)_max_ < 0.001
279 parameters	Δρ_max_ = 0.64 e Å^−^^3^
0 restraints	Δρ_min_ = −0.78 e Å^−^^3^

### Special details

**Table:**

Geometry. All e.s.d.'s (except the e.s.d. in the dihedral angle between two l.s. planes) are estimated using the full covariance matrix. The cell e.s.d.'s are taken into account individually in the estimation of e.s.d.'s in distances, angles and torsion angles; correlations between e.s.d.'s in cell parameters are only used when they are defined by crystal symmetry. An approximate (isotropic) treatment of cell e.s.d.'s is used for estimating e.s.d.'s involving l.s. planes.
Refinement. Refinement of *F*^2^ against ALL reflections. The weighted *R*-factor *wR* and goodness of fit *S* are based on *F*^2^, conventional *R*-factors *R* are based on *F*, with *F* set to zero for negative *F*^2^. The threshold expression of *F*^2^ > σ(*F*^2^) is used only for calculating *R*-factors(gt) *etc*. and is not relevant to the choice of reflections for refinement. *R*-factors based on *F*^2^ are statistically about twice as large as those based on *F*, and *R*- factors based on ALL data will be even larger.

### Fractional atomic coordinates and isotropic or equivalent isotropic displacement parameters (Å^2^)

**Table:**

	*x*	*y*	*z*	*U*_iso_*/*U*_eq_	Occ. (<1)
Ni	0.5000	0.5000	0.5000	0.02039 (14)	
S1	0.38649 (8)	0.38353 (7)	0.64197 (5)	0.02552 (17)	
S2	0.67986 (8)	0.29788 (6)	0.46088 (5)	0.02452 (16)	
P1	0.55031 (9)	0.24313 (7)	0.58030 (6)	0.02530 (17)	
O1	0.6644 (3)	0.15123 (18)	0.67162 (16)	0.0295 (4)	
N1	0.6658 (3)	0.5003 (2)	0.61646 (17)	0.0205 (4)	
N2	0.9368 (3)	0.5058 (2)	0.96412 (18)	0.0256 (5)	
C1	0.7711 (4)	0.1916 (3)	0.7302 (2)	0.0309 (6)	
H1A	0.7063	0.2423	0.7859	0.037*	
H1B	0.8205	0.2396	0.6782	0.037*	
C2	0.9051 (4)	0.0857 (3)	0.7856 (2)	0.0303 (6)	
H2	0.9674	0.0343	0.7286	0.036*	
C3	1.0219 (4)	0.1321 (3)	0.8386 (3)	0.0420 (8)	
H3A	1.1097	0.0648	0.8754	0.063*	
H3B	0.9610	0.1855	0.8923	0.063*	
H3C	1.0695	0.1755	0.7818	0.063*	
C4	0.8358 (5)	0.0118 (3)	0.8709 (3)	0.0399 (8)	
H4A	0.9260	−0.0558	0.9049	0.060*	
H4B	0.7624	−0.0175	0.8352	0.060*	
H4C	0.7744	0.0613	0.9273	0.060*	
O2	0.4688 (3)	0.1558 (2)	0.54352 (18)	0.0365 (5)	0.50
C5	0.3314 (8)	0.2219 (5)	0.4584 (5)	0.0237 (12)	0.50
H5A	0.2908	0.3076	0.4683	0.028*	0.50
H5B	0.3821	0.2143	0.3842	0.028*	0.50
C6	0.2185 (9)	0.1915 (8)	0.4606 (6)	0.0447 (17)	0.50
H6A	0.1330	0.2627	0.4881	0.054*	0.50
C7	0.1788 (5)	0.0918 (4)	0.5366 (3)	0.0565 (11)	0.50
H7A	0.0721	0.0903	0.5173	0.085*	0.50
H7B	0.1752	0.1075	0.6131	0.085*	0.50
H7C	0.2640	0.0148	0.5273	0.085*	0.50
C8	0.1386 (4)	0.1940 (3)	0.3445 (3)	0.0392 (7)	0.50
H8A	0.0449	0.1669	0.3574	0.059*	0.50
H8B	0.2210	0.1413	0.2985	0.059*	0.50
H8C	0.1017	0.2754	0.3073	0.059*	0.50
O32	0.4688 (3)	0.1558 (2)	0.54352 (18)	0.0365 (5)	0.50
C35	0.3937 (10)	0.1619 (8)	0.4454 (5)	0.0359 (15)	0.50
H35A	0.3574	0.2468	0.4149	0.043*	0.50
H35B	0.4830	0.1191	0.3961	0.043*	0.50
C36	0.2674 (7)	0.1257 (5)	0.4299 (5)	0.0216 (10)	0.50
H36A	0.3227	0.0475	0.4005	0.026*	0.50
C37	0.1788 (5)	0.0918 (4)	0.5366 (3)	0.0565 (11)	0.50
H37A	0.0915	0.0649	0.5169	0.085*	0.50
H37B	0.1312	0.1616	0.5767	0.085*	0.50
H37C	0.2582	0.0278	0.5830	0.085*	0.50
C38	0.1386 (4)	0.1940 (3)	0.3445 (3)	0.0392 (7)	0.50
H38A	0.0580	0.1546	0.3438	0.059*	0.50
H38B	0.1916	0.1960	0.2720	0.059*	0.50
H38C	0.0833	0.2755	0.3627	0.059*	0.50
C9	0.6097 (3)	0.5338 (2)	0.7157 (2)	0.0226 (5)	
H9	0.4946	0.5601	0.7293	0.027*	
C10	0.7099 (3)	0.5319 (2)	0.7990 (2)	0.0227 (5)	
H10	0.6648	0.5576	0.8676	0.027*	
C11	0.8794 (3)	0.4915 (2)	0.7802 (2)	0.0214 (5)	
C12	0.9386 (3)	0.4578 (2)	0.6774 (2)	0.0224 (5)	
H12	1.0532	0.4305	0.6617	0.027*	
C13	0.8285 (3)	0.4644 (2)	0.5982 (2)	0.0225 (5)	
H13	0.8699	0.4425	0.5278	0.027*	
C14	0.9924 (3)	0.4844 (2)	0.8672 (2)	0.0236 (5)	
H14	1.1066	0.4638	0.8516	0.028*	
C15	0.5105 (4)	0.7783 (3)	−0.0282 (3)	0.0331 (7)	
C16	0.4464 (4)	0.6899 (3)	0.0108 (3)	0.0341 (7)	
H16	0.4841	0.6424	0.0782	0.041*	
C17	0.3289 (4)	0.6702 (3)	−0.0467 (3)	0.0396 (7)	
H17	0.2853	0.6104	−0.0182	0.048*	
C18	0.2746 (4)	0.7376 (3)	−0.1460 (3)	0.0412 (8)	
H18	0.1939	0.7242	−0.1858	0.049*	
C19	0.3388 (5)	0.8241 (3)	−0.1867 (3)	0.0409 (8)	
H19	0.3024	0.8700	−0.2550	0.049*	
C20	0.4562 (4)	0.8450 (3)	−0.1289 (3)	0.0374 (7)	
H20	0.4995	0.9048	−0.1578	0.045*	
C21	0.6361 (5)	0.8005 (3)	0.0363 (3)	0.0461 (8)	
H21A	0.6290	0.7699	0.1134	0.069*	
H21B	0.6150	0.8862	0.0304	0.069*	
H21C	0.7460	0.7598	0.0069	0.069*	

### Atomic displacement parameters (Å^2^)

**Table:**

	*U*^11^	*U*^22^	*U*^33^	*U*^12^	*U*^13^	*U*^23^
Ni	0.0115 (2)	0.0342 (3)	0.0152 (2)	−0.00714 (19)	−0.00478 (17)	−0.00191 (18)
S1	0.0150 (3)	0.0399 (4)	0.0199 (3)	−0.0084 (3)	−0.0028 (2)	0.0008 (3)
S2	0.0158 (3)	0.0365 (4)	0.0203 (3)	−0.0070 (3)	−0.0042 (2)	−0.0037 (3)
P1	0.0194 (3)	0.0345 (4)	0.0219 (3)	−0.0092 (3)	−0.0064 (3)	−0.0007 (3)
O1	0.0293 (11)	0.0306 (10)	0.0278 (10)	−0.0096 (9)	−0.0100 (9)	0.0017 (8)
N1	0.0135 (10)	0.0313 (11)	0.0171 (10)	−0.0078 (9)	−0.0058 (8)	−0.0010 (8)
N2	0.0194 (11)	0.0366 (12)	0.0227 (11)	−0.0107 (10)	−0.0096 (9)	−0.0029 (9)
C1	0.0312 (15)	0.0320 (14)	0.0269 (14)	−0.0061 (12)	−0.0147 (12)	−0.0020 (11)
C2	0.0295 (15)	0.0310 (14)	0.0238 (13)	−0.0015 (12)	−0.0073 (12)	−0.0019 (11)
C3	0.0416 (19)	0.0414 (17)	0.0380 (17)	−0.0051 (15)	−0.0229 (15)	−0.0037 (14)
C4	0.0437 (19)	0.0377 (16)	0.0264 (15)	−0.0022 (14)	−0.0030 (14)	0.0049 (13)
O2	0.0376 (12)	0.0486 (13)	0.0326 (11)	−0.0256 (11)	−0.0088 (10)	−0.0040 (10)
C5	0.020 (3)	0.016 (2)	0.031 (3)	−0.001 (2)	−0.017 (2)	0.003 (2)
C6	0.032 (4)	0.058 (5)	0.043 (4)	−0.019 (4)	−0.015 (3)	0.015 (4)
C7	0.045 (2)	0.084 (3)	0.049 (2)	−0.042 (2)	−0.0094 (18)	0.022 (2)
C8	0.0304 (16)	0.0492 (18)	0.0411 (18)	−0.0182 (15)	−0.0093 (14)	0.0007 (15)
O32	0.0376 (12)	0.0486 (13)	0.0326 (11)	−0.0256 (11)	−0.0088 (10)	−0.0040 (10)
C35	0.037 (4)	0.053 (5)	0.027 (3)	−0.028 (4)	−0.006 (3)	0.001 (3)
C36	0.024 (3)	0.016 (2)	0.027 (3)	−0.007 (2)	−0.001 (2)	−0.008 (2)
C37	0.045 (2)	0.084 (3)	0.049 (2)	−0.042 (2)	−0.0094 (18)	0.022 (2)
C38	0.0304 (16)	0.0492 (18)	0.0411 (18)	−0.0182 (15)	−0.0093 (14)	0.0007 (15)
C9	0.0167 (12)	0.0315 (13)	0.0190 (12)	−0.0071 (10)	−0.0057 (10)	−0.0022 (10)
C10	0.0190 (12)	0.0303 (13)	0.0192 (12)	−0.0082 (10)	−0.0061 (10)	−0.0024 (10)
C11	0.0174 (12)	0.0281 (12)	0.0204 (12)	−0.0098 (10)	−0.0075 (10)	0.0000 (10)
C12	0.0135 (11)	0.0305 (13)	0.0231 (12)	−0.0080 (10)	−0.0041 (10)	0.0001 (10)
C13	0.0145 (12)	0.0324 (13)	0.0207 (12)	−0.0080 (10)	−0.0035 (10)	−0.0019 (10)
C14	0.0188 (12)	0.0301 (13)	0.0239 (12)	−0.0102 (10)	−0.0075 (10)	−0.0020 (10)
C15	0.0297 (15)	0.0316 (14)	0.0328 (16)	−0.0039 (12)	0.0015 (13)	−0.0061 (12)
C16	0.0316 (16)	0.0323 (14)	0.0305 (15)	−0.0035 (12)	0.0062 (13)	−0.0006 (12)
C17	0.0360 (17)	0.0420 (17)	0.0404 (18)	−0.0140 (14)	0.0092 (14)	−0.0067 (14)
C18	0.0318 (17)	0.0471 (18)	0.0415 (18)	−0.0079 (15)	0.0004 (14)	−0.0119 (15)
C19	0.0439 (19)	0.0386 (16)	0.0303 (16)	−0.0030 (15)	−0.0023 (14)	−0.0010 (13)
C20	0.0431 (18)	0.0309 (15)	0.0340 (16)	−0.0091 (14)	0.0030 (14)	−0.0010 (13)
C21	0.046 (2)	0.0434 (18)	0.050 (2)	−0.0162 (16)	−0.0097 (17)	−0.0032 (16)

### Geometric parameters (Å, °)

**Table:**

Ni—N1	2.096 (2)	C8—H8C	0.9800
Ni—N1^i^	2.096 (2)	O32—C35	1.388 (7)
Ni—S1^i^	2.4806 (8)	C35—C36	1.352 (8)
Ni—S1	2.4806 (8)	C35—H35A	0.9900
Ni—S2	2.4823 (8)	C35—H35B	0.9900
Ni—S2^i^	2.4823 (8)	C36—C38	1.519 (6)
S1—P1	1.9949 (11)	C36—C37	1.564 (7)
S2—P1	1.9864 (10)	C36—H36A	1.0000
P1—O32	1.585 (2)	C37—H37A	0.9800
P1—O2	1.585 (2)	C37—H37B	0.9800
P1—O1	1.587 (2)	C37—H37C	0.9800
O1—C1	1.453 (3)	C38—H38A	0.9800
N1—C13	1.342 (3)	C38—H38B	0.9800
N1—C9	1.344 (3)	C38—H38C	0.9800
N2—C14	1.281 (4)	C9—C10	1.379 (3)
N2—N2^ii^	1.410 (4)	C9—H9	0.9500
C1—C2	1.511 (4)	C10—C11	1.396 (4)
C1—H1A	0.9900	C10—H10	0.9500
C1—H1B	0.9900	C11—C12	1.391 (4)
C2—C4	1.525 (4)	C11—C14	1.472 (3)
C2—C3	1.533 (4)	C12—C13	1.384 (3)
C2—H2	1.0000	C12—H12	0.9500
C3—H3A	0.9800	C13—H13	0.9500
C3—H3B	0.9800	C14—H14	0.9500
C3—H3C	0.9800	C15—C16	1.393 (4)
C4—H4A	0.9800	C15—C20	1.398 (4)
C4—H4B	0.9800	C15—C21	1.505 (5)
C4—H4C	0.9800	C16—C17	1.381 (5)
O2—C5	1.558 (6)	C16—H16	0.9500
C5—C6	1.164 (9)	C17—C18	1.387 (5)
C5—H5A	0.9900	C17—H17	0.9500
C5—H5B	0.9900	C18—C19	1.380 (5)
C6—C7	1.553 (8)	C18—H18	0.9500
C6—C8	1.617 (8)	C19—C20	1.389 (5)
C6—H6A	1.0000	C19—H19	0.9500
C7—H7A	0.9800	C20—H20	0.9500
C7—H7B	0.9800	C21—H21A	0.9800
C7—H7C	0.9800	C21—H21B	0.9800
C8—H8A	0.9800	C21—H21C	0.9800
C8—H8B	0.9800		
			
N1—Ni—N1^i^	180.0	H7B—C7—H7C	109.5
N1—Ni—S1^i^	91.71 (6)	C6—C8—H8A	109.5
N1^i^—Ni—S1^i^	88.29 (6)	C6—C8—H8B	109.5
N1—Ni—S1	88.29 (6)	H8A—C8—H8B	109.5
N1^i^—Ni—S1	91.71 (6)	C6—C8—H8C	109.5
S1^i^—Ni—S1	180.00 (3)	H8A—C8—H8C	109.5
N1—Ni—S2	90.35 (6)	H8B—C8—H8C	109.5
N1^i^—Ni—S2	89.65 (6)	C35—O32—P1	129.0 (3)
S1^i^—Ni—S2	98.04 (3)	C36—C35—O32	127.6 (6)
S1—Ni—S2	81.96 (3)	C36—C35—H35A	105.4
N1—Ni—S2^i^	89.65 (6)	O32—C35—H35A	105.4
N1^i^—Ni—S2^i^	90.35 (6)	C36—C35—H35B	105.4
S1^i^—Ni—S2^i^	81.96 (3)	O32—C35—H35B	105.4
S1—Ni—S2^i^	98.04 (3)	H35A—C35—H35B	106.0
S2—Ni—S2^i^	180.0	C35—C36—C38	120.7 (5)
P1—S1—Ni	84.11 (3)	C35—C36—C37	115.7 (5)
P1—S2—Ni	84.24 (3)	C38—C36—C37	108.6 (4)
O32—P1—O2	0.00 (17)	C35—C36—H36A	103.1
O32—P1—O1	96.75 (12)	C38—C36—H36A	103.1
O2—P1—O1	96.75 (12)	C37—C36—H36A	103.1
O32—P1—S2	113.02 (9)	C36—C37—H37A	109.5
O2—P1—S2	113.02 (9)	C36—C37—H37B	109.5
O1—P1—S2	111.99 (9)	H37A—C37—H37B	109.5
O32—P1—S1	112.44 (10)	C36—C37—H37C	109.5
O2—P1—S1	112.44 (10)	H37A—C37—H37C	109.5
O1—P1—S1	112.53 (9)	H37B—C37—H37C	109.5
S2—P1—S1	109.66 (5)	C36—C38—H38A	109.5
C1—O1—P1	117.69 (18)	C36—C38—H38B	109.5
C13—N1—C9	117.5 (2)	H38A—C38—H38B	109.5
C13—N1—Ni	122.93 (18)	C36—C38—H38C	109.5
C9—N1—Ni	119.51 (17)	H38A—C38—H38C	109.5
C14—N2—N2^ii^	111.6 (3)	H38B—C38—H38C	109.5
O1—C1—C2	109.9 (2)	N1—C9—C10	123.6 (2)
O1—C1—H1A	109.7	N1—C9—H9	118.2
C2—C1—H1A	109.7	C10—C9—H9	118.2
O1—C1—H1B	109.7	C9—C10—C11	118.5 (2)
C2—C1—H1B	109.7	C9—C10—H10	120.8
H1A—C1—H1B	108.2	C11—C10—H10	120.8
C1—C2—C4	111.8 (3)	C12—C11—C10	118.4 (2)
C1—C2—C3	108.0 (3)	C12—C11—C14	120.9 (2)
C4—C2—C3	110.8 (3)	C10—C11—C14	120.8 (2)
C1—C2—H2	108.7	C13—C12—C11	119.1 (2)
C4—C2—H2	108.7	C13—C12—H12	120.4
C3—C2—H2	108.7	C11—C12—H12	120.4
C2—C3—H3A	109.5	N1—C13—C12	122.9 (2)
C2—C3—H3B	109.5	N1—C13—H13	118.6
H3A—C3—H3B	109.5	C12—C13—H13	118.6
C2—C3—H3C	109.5	N2—C14—C11	119.9 (2)
H3A—C3—H3C	109.5	N2—C14—H14	120.0
H3B—C3—H3C	109.5	C11—C14—H14	120.0
C2—C4—H4A	109.5	C16—C15—C20	118.4 (3)
C2—C4—H4B	109.5	C16—C15—C21	120.7 (3)
H4A—C4—H4B	109.5	C20—C15—C21	120.9 (3)
C2—C4—H4C	109.5	C17—C16—C15	121.2 (3)
H4A—C4—H4C	109.5	C17—C16—H16	119.4
H4B—C4—H4C	109.5	C15—C16—H16	119.4
C5—O2—P1	111.3 (3)	C16—C17—C18	120.0 (3)
C6—C5—O2	117.8 (6)	C16—C17—H17	120.0
C6—C5—H5A	107.9	C18—C17—H17	120.0
O2—C5—H5A	107.9	C19—C18—C17	119.5 (3)
C6—C5—H5B	107.9	C19—C18—H18	120.2
O2—C5—H5B	107.9	C17—C18—H18	120.2
H5A—C5—H5B	107.2	C18—C19—C20	120.8 (3)
C5—C6—C7	130.3 (6)	C18—C19—H19	119.6
C5—C6—C8	117.4 (6)	C20—C19—H19	119.6
C7—C6—C8	104.4 (5)	C19—C20—C15	120.1 (3)
C5—C6—H6A	99.3	C19—C20—H20	120.0
C7—C6—H6A	99.3	C15—C20—H20	120.0
C8—C6—H6A	99.3	C15—C21—H21A	109.5
C6—C7—H7A	109.5	C15—C21—H21B	109.5
C6—C7—H7B	109.5	H21A—C21—H21B	109.5
H7A—C7—H7B	109.5	C15—C21—H21C	109.5
C6—C7—H7C	109.5	H21A—C21—H21C	109.5
H7A—C7—H7C	109.5	H21B—C21—H21C	109.5
			
N1—Ni—S1—P1	−91.85 (7)	O32—P1—O2—C5	0(43)
N1^i^—Ni—S1—P1	88.15 (7)	O1—P1—O2—C5	174.5 (3)
S1^i^—Ni—S1—P1	114 (100)	S2—P1—O2—C5	−68.1 (3)
S2—Ni—S1—P1	−1.26 (3)	S1—P1—O2—C5	56.7 (3)
S2^i^—Ni—S1—P1	178.74 (3)	P1—O2—C5—C6	−146.3 (7)
N1—Ni—S2—P1	89.49 (7)	O2—C5—C6—C7	−0.2 (15)
N1^i^—Ni—S2—P1	−90.51 (7)	O2—C5—C6—C8	−143.8 (5)
S1^i^—Ni—S2—P1	−178.74 (3)	O2—P1—O32—C35	0(59)
S1—Ni—S2—P1	1.26 (3)	O1—P1—O32—C35	−158.0 (5)
S2^i^—Ni—S2—P1	16 (100)	S2—P1—O32—C35	−40.6 (5)
Ni—S2—P1—O32	124.67 (10)	S1—P1—O32—C35	84.2 (5)
Ni—S2—P1—O2	124.67 (10)	P1—O32—C35—C36	−147.9 (7)
Ni—S2—P1—O1	−127.32 (9)	O32—C35—C36—C38	144.5 (7)
Ni—S2—P1—S1	−1.65 (4)	O32—C35—C36—C37	10.4 (12)
Ni—S1—P1—O32	−124.99 (10)	C13—N1—C9—C10	−0.6 (4)
Ni—S1—P1—O2	−124.99 (10)	Ni—N1—C9—C10	177.1 (2)
Ni—S1—P1—O1	127.01 (9)	N1—C9—C10—C11	−1.0 (4)
Ni—S1—P1—S2	1.65 (4)	C9—C10—C11—C12	1.5 (4)
O32—P1—O1—C1	178.8 (2)	C9—C10—C11—C14	−178.2 (2)
O2—P1—O1—C1	178.8 (2)	C10—C11—C12—C13	−0.5 (4)
S2—P1—O1—C1	60.6 (2)	C14—C11—C12—C13	179.2 (2)
S1—P1—O1—C1	−63.5 (2)	C9—N1—C13—C12	1.7 (4)
N1^i^—Ni—N1—C13	83 (100)	Ni—N1—C13—C12	−175.9 (2)
S1^i^—Ni—N1—C13	−52.9 (2)	C11—C12—C13—N1	−1.1 (4)
S1—Ni—N1—C13	127.1 (2)	N2^ii^—N2—C14—C11	179.8 (3)
S2—Ni—N1—C13	45.1 (2)	C12—C11—C14—N2	−173.9 (3)
S2^i^—Ni—N1—C13	−134.9 (2)	C10—C11—C14—N2	5.8 (4)
N1^i^—Ni—N1—C9	−95 (100)	C20—C15—C16—C17	−1.6 (5)
S1^i^—Ni—N1—C9	129.46 (19)	C21—C15—C16—C17	178.8 (3)
S1—Ni—N1—C9	−50.54 (19)	C15—C16—C17—C18	1.0 (5)
S2—Ni—N1—C9	−132.48 (19)	C16—C17—C18—C19	0.0 (5)
S2^i^—Ni—N1—C9	47.52 (19)	C17—C18—C19—C20	−0.5 (5)
P1—O1—C1—C2	−162.1 (2)	C18—C19—C20—C15	−0.1 (5)
O1—C1—C2—C4	−62.2 (3)	C16—C15—C20—C19	1.1 (5)
O1—C1—C2—C3	175.6 (3)	C21—C15—C20—C19	−179.3 (3)

Symmetry codes: (i) −*x*+1, −*y*+1, −*z*+1; (ii) −*x*+2, −*y*+1, −*z*+2.

### Hydrogen-bond geometry (Å, °)

**Table:**

*D*—H···*A*	*D*—H	H···*A*	*D*···*A*	*D*—H···*A*
C12—H12···S1^iii^	0.95	2.76	3.694 (3)	169

Symmetry codes: (iii) *x*+1, *y*, *z*.
